# An untargeted metabolome-wide association study of maternal perinatal tobacco smoking in newborn blood spots

**DOI:** 10.1007/s11306-025-02225-3

**Published:** 2025-02-20

**Authors:** Di He, Qi Yan, Karan Uppal, Douglas I. Walker, Dean P. Jones, Beate Ritz, Julia E. Heck

**Affiliations:** 1 Department of Epidemiology, Fielding School of Public Health, 650 Charles E. Young Drive, Box 951772, Los Angeles, CA 90095-1772 USA; 2 Division of Pulmonary, Allergy and Critical Care Medicine, Clinical Biomarkers Laboratory, School of Medicine, Emory University, Atlanta, GA USA; 3 Gangarosa Department of Environmental Health, Rollins School of Public Health, Emory University, Atlanta, GA USA; 4 Department of Medicine, Emory University, Atlanta, GA USA; 5 College of Health and Public Service, University of North Texas, Denton, TX USA

**Keywords:** Maternal smoking, Child outcomes, High-resolution metabolomics, Inflammatory responses, Neonatal blood spots

## Abstract

**Introduction:**

Maternal tobacco smoking in the perinatal period increases the risk for adverse outcomes in offspring.

**Objective:**

To better understand the biological pathways through which maternal tobacco use may have long-term impacts on child metabolism, we performed a high-resolution metabolomics (HRM) analysis in newborns, following an untargeted metabolome-wide association study workflow.

**Methods:**

The study population included 899 children without cancer diagnosis before age 6 and born between 1983 and 2011 in California. Newborn dried blood spots were collected by the California Genetic Disease Screening Program between 12 and 48 h after birth and stored for later research use. Based on HRM, we considered mothers to be active smokers if they were self- or provider-reported smokers on birth certificates or if we detected any cotinine or high hydroxycotinine intensities in newborn blood. We used partial least squares discriminant analysis and Mummichog pathway analysis to identify metabolites and metabolic pathways associated with maternal tobacco smoking.

**Results:**

A total of 26,183 features were detected with HRM, including 1003 that were found to be associated with maternal smoking late in pregnancy and early postpartum (Variable Importance in Projection (VIP) scores > = 2). Smoking affected metabolites and metabolic pathways in neonatal blood including vitamin A (retinol) metabolism, the kynurenine pathway, and tryptophan and arachidonic acid metabolism.

**Conclusion:**

The smoking-associated metabolites and pathway perturbations that we identified suggested inflammatory responses and have also been implicated in chronic diseases of the central nervous system and the lung. Our results suggest that infant metabolism in the early postnatal period reflects smoking specific physiologic responses to maternal smoking with strong biologic plausibility.

**Supplementary Information:**

The online version contains supplementary material available at 10.1007/s11306-025-02225-3.

## Introduction

Tobacco use is one of the most significant public health issues globally, with the number of global consumers of tobacco increasing over the past few decades (Saleheen et al., 2014). In the U.S., the prevalence of current cigarette smoking among adults declined from 51% in 1965 to 31% in 2012 among males and from 34 to 23% among females (US Department of Health and Human Services, 2014). It has also been reported that approximately 50% of California women smoked during pregnancy in the early 1980s (Keyes et al., 2013) and this number declined to 15% in the 1990s (Mahadevan et al., 2007) and 5% in the 2010s (Sun et al., 2022). Despite the rate declining by more than half since 1964, cigarette smoking remains the most preventable cause of disease and death in the U.S. with disparities persisting across population groups (Centers for Disease Control and Prevention, 2018). In recent decades, smoking rates in California pregnant women have been approximately 5% lower than nationwide (California Department of Public Health, 2014), which is partly due to demographics as immigrant Mexican women make up about 25% of all pregnant women and most are non-smokers (Hoggatt et al., 2012). Components in cigarette smoke including polycyclic aromatic hydrocarbons (PAHs), N-nitrosamines, aromatic amines, aldehydes, volatile organic hydrocarbons, and metals have long been known or suspected carcinogens. Nicotine has been shown in various studies to stimulate the growth of cancer cells and the proliferation of endothelial cells in vivo, indicating its potential role in advancing the progression of pre-existing tumors (Catassi et al., 2008). Therefore, nicotine may be contributing to childhood cancers due to prenatal and early childhood exposure from smoking mothers. Smoking in pregnancy has also been associated with adverse infant and child outcomes, including low birth weight, preterm birth, and congenital anomalies (Hackshaw et al., 2011; Pollack et al., 2000).

For pregnant women close to the time of delivery, cotinine levels in their newborns’ dried blood spots are a reliable biomarker of maternal smoking with high sensitivity (92.3%) and specificity (99.7%) (Bardy et al., 1993; Yang et al., 2013). Cotinine best represents recent smoking (Koskela et al., 2000), with a half-life longer than its parent compound, nicotine, and it is detectable for 15–20 h after active smoking (Murphy et al., 2013; Wall et al., 1988). Hydroxycotinine, metabolized by the hepatic enzyme cytochrome P450 2A6, is the major metabolite of cotinine in most individuals (Jacob et al., 2011; Murphy et al., 2013), and is detectable for a longer period with an approximated half-life of 18 h (Dempsey et al., 2000). Archived dried blood spots (DBS), routinely collected by state registries and stored for decades, provide a unique resource for investigating maternal behaviors, exposures, and rare childhood diseases through metabolic profiling, including studies on maternal smoking (He et al., 2023b). These findings highlight the utility of cotinine and its metabolites as robust biomarkers for confirming maternal smoking during pregnancy, especially when using newborn dried blood spots.

Tobacco smoking is related to metabolomic changes as recently documented for adult smokers, children exposed to passive smoke, and pregnant women (Fischer et al., 2017; Jones et al., 2016; Masvosva et al., 2024; Tan et al., 2022; Zhu et al., 2021). A study of military personnel observed alterations in lipid and xenobiotic metabolism, and diverse effects on amino acid, sialic acid and purine and pyrimidine metabolism in tobacco smokers compared to non-smokers (Jones et al., 2016). Another study among preschool children reported secondhand smoke-induced urinary metabolites including kynurenine, tyrosyl-tryptophan, and 1-(3-pyridinyl)-1,4-butanediol, peptides, and pyridines (Zhu et al., 2021). Utilizing second-trimester amniotic fluid, a third study found low-level maternal nicotine exposure from light smoking or secondhand smoke to be associated with dysregulated metabolic pathways in the fetus such as aspartate and asparagine metabolism, pyrimidine metabolism, and metabolism of other amino acids, and also saw decreases in acetylated polyamines (Fischer et al., 2017). The same research team later examined how smoking during pregnancy was associated with adverse birth outcomes among African American women using maternal urine and blood samples collected during pregnancy (Tan et al., 2022). Recently, a study approached this question by employing an untargeted metabolomics analysis in pregnant women’s first trimester plasma and identified metabolites associated with maternal smoking that indicated endocrine disruption, oxidative stress, and lipid metabolism (Masvosva et al., 2024). Over the years, researchers also started to assess maternal smoking exposure and neonatal metabolic outcomes using biospecimens from offspring. Two studies assessed maternal smoking and the impact on child metabolic outcomes in the cord blood of newborns using a targeted metabolomics approach, focusing on a selected set of metabolites (Cajachagua-Torres et al., 2022; Rolle-Kampczyk et al., 2016).

To the best of our knowledge, there have been no studies investigating maternal smoking-associated metabolic alterations in offspring using an untargeted profiling approach among neonatal biospecimens. To address the current research gaps and better understand the biological pathways through which maternal late pregnancy smoking may be increasing the risk of disease, we performed a high-resolution metabolomics (HRM) analysis of 899 children born from 1983 to 2011 in California following an untargeted metabolome-wide association study (MWAS) workflow.

## Methods

### Study population

The study utilized the controls from a population-based case control study of childhood cancers (Heck et al., 2012) that enrolled 1400 children with cases ascertained from the California Cancer Registry. Population controls (20:1 matching rate) were randomly selected from California birth rolls and frequency-matched to cases by birth year. For this analysis, we only included 899 cancer-free controls (Ritz et al., 2022). Detailed demographic, socioeconomic, and gestational information for each child was collected from California birth certificates. In the years from 1989 to 2005 medical providers collected limited smoking information during pregnancy by answering the question “Were there pregnancy complications due to tobacco use during pregnancy?” Starting in 2007, the “number of cigarettes per day (3 months before pregnancy and during each trimester),” was reported on the California birth certificate (Heck et al., 2016).

We obtained neonatal dried blood spots (DBS) for this analysis from the California Genetic Disease Screening Program. Nearly all newborns in the United States participate in a newborn screening program for genetic and metabolic diseases, and in California, the DBS are stored for research purposes after testing is complete (Gonzales, 2011). Blood samples are collected from newborns by a heel-stick from the child between 12 and 48 h after birth. The blood fills six small circles on specialized filter paper and is dried at room temperature for at least 3 h before shipment to the Neonatal and Prenatal Screening Laboratory within 24 h of collection (California Department of Public Health, 2023). Since 1982, specimens left-over after routine screening are packed and stored at − 20 °C. Additional details concerning dried blood specimen collection and storage are described elsewhere (Yang et al., 2013).

### High-resolution metabolomics

Neonatal blood spots were analyzed using liquid chromatography with ultra-high resolution mass spectrometry (LC-HRMS; Fusion, Thermo Scientific) (Liu et al., 2020a). Samples were punched using a 5 mm hole puncher and treated with 2:1 acetonitrile in water containing a mixture of stable isotopic internal standards. Samples were mixed for 12 h at 0–4 °C in the dark and then centrifuged to remove particulate matter. The resulting supernatant was analyzed in triplicate using hydrophilic interaction liquid chromatography (HILIC) with positive electrospray ionization (ESI) and C18 hydrophobic reversed-phase chromatography with negative ESI to enhance the coverage of metabolic feature detection (Liang et al., 2018). The mass spectrometer was operated using ESI mode at a resolution of 120,000 and mass-to-charge ratio (*m/z*) range of 85–1275. Blood spot samples were analyzed in batches of 40. To evaluate system performance, we used two separate quality assessment methods. Our first Q.C. sample was NIST 1950 (Simon-Manso et al., 2013), which was analyzed at the beginning and end of the entire analytical run. The second Q.C. sample (Q-Std) included commercially purchased plasma pooled from an unknown number of males and females. Q-Std was analyzed at the beginning, middle, and end of each batch of 40 samples for normalization and batch effect evaluation. Raw data files were extracted and aligned using *apLCMS* (Yu et al., 2009) with modifications by *xMSanalyzer* (Uppal et al., 2013). Uniquely detected ions consisted of mass-to-charge ratio (*m/z*), retention time (rt), and ion abundance, referred to as metabolite features. Prior to data analysis, metabolite features were batch corrected using wavelet analysis (Deng et al., 2019). For this analysis, we only included metabolic features with fewer than 30% missing values across all samples, with median coefficients of variation (CV) among technical replicates < 30% and Pearson correlation > 0.7 (Go et al., 2015a). Following quality assessment, replicate intensities were summarized using the median value, log2 transformed, and auto-scaled. Missing values were imputed using k-nearest neighbors (k = 10) (Troyanskaya et al., 2001) imputed using the *impute* R package.

### Exposure assessment

We extracted cotinine (*m/z* = 177.1023) and hydroxycotinine (*m/z* = 193.0973) from the HRM feature table (identification confidence level 1). The definition of active smokers was described in detail in our previous study (He et al., 2023a). In short, contemporaneous surveys of California women indicate a decline in maternal smoking during pregnancy, with prevalence decreasing from approximately 50% in 1980 to 14% between 1995 and 2002, and further to 5% from 2008 to 2018. Hydroxycotinine, detected in 20% of samples, can originate from sources other than smoking. Therefore, consistent with the reported prevalence trends, we classified mothers as smokers if hydroxycotinine intensities in newborn blood were within the top 14%, if the newborn blood had cotinine detected, or if they were self- or provider reported smokers on birth certificates. Overall, this yielded an average prevalence of smoking of 17% across the study period from any of the smoking indicators.

We previously examined agreement among these different smoking metrics (He et al., 2023a). Among women provider-reported smokers on birth records, 65% had cotinine detected in their newborn blood and 55% hydroxycotinine. Cotinine and hydroxycotinine were also present in 78 and 56%, respectively, of blood spots of newborns born to mothers were self-reported smokers. We did not attempt to stratify the extent of second-hand smoking exposure due to the lack of larger volume blood spots that would be needed to reach the detection limit for these metabolites (Yang et al., 2013). Out of mothers of the 883 cancer-free controls, 153 smoked in pregnancy, as measured by any of the smoking indicators: 17 (1.9%) according to self or provider reported smoking during pregnancy, 62 (7.0%) according to cotinine, and 92 (10.4%) according to hydroxycotinine.

### Statistical analysis

After excluding 6 samples considered outliers during the high-resolution metabolomics process due to sample quality issues and 10 samples with missing covariates, a total of 883 samples were left in the analysis. To control for potential confounding from maternal race/ethnicity (White non-Hispanic, Hispanic of any race, other), maternal age (< 20, 20–24, 25–29, 30–34, > = 35), birth year (1983–1990, 1991–2000, 2001–2011), infant’s sex (male, female), and neighborhood socioeconomic status (SES, categorical levels 1–5) (Yost et al., 2001), we regressed the intensities of all metabolites other than cotinine and hydroxycotinine against confounders listed above in the following analyses.

We used a combination of univariate and multivariate analyses to identify neonatal blood spot metabolic features associated with maternal perinatal smoking. We adopted multivariate analysis partial least square—discriminant analysis (PLS-DA) to identify features that differentiate smokers from non-smokers (Gromski et al., 2015; Liland, 2011). Features with Variable Importance in Projection (VIP) scores > = 2 were selected and fold changes for metabolites were calculated as the ratio of covariate-adjusted intensities comparing smokers and non-smokers. Score plots were used to visualize how the PLS-DA analysis effectively differentiates features by smoking status. Logistic regression was then used to assess associations between discriminative metabolite features and maternal smoking. All feature selection approaches were implemented within the R package *mixOmics* v6.3.1. We adjusted for multiple testing using false-discovery rate (FDR)-adjusted *p*-values.

Discriminative features selected by PLS-DA were first matched to a reference database of chemical standards (identification confidence level 1) previously analyzed using the same HRM platform (Liu et al., 2020b; Schymanski et al., 2014). The error tolerance was ± 5 parts-per-million (ppm) and ± 15 s (s) for *m/z* and retention time, respectively. Details about the reference database have been published previously (Go et al., 2015b; Liu et al., 2020a). Additional features were annotated by *xMSannotator* (Uppal et al., 2017) which links to databases of metabolites (Human Metabolome Database; LipidMaps; KEGG; others). Accurate mass *m/z* for adducts formed under positive/negative ESI mode were matched to the Human Metabolome Database (HMDB), with a mass error threshold of 10 ppm. *xMSannotator* uses a scoring system (0–3, a higher score representing a higher-confidence result) based upon correlation modularity clustering combined with isotopic, adduct, and mass defect grouping to improve the annotation of high-resolution mass spectrometry data. Only results with an annotation score > 2 were kept (idenfication confidence level 4) (Schymanski et al., 2014).

In order to identify perturbed metabolism pathways, we conducted pathway enrichment analysis utilizing *mummichog* v 2.4.2 (Bonvallot et al., 2013). Mummichog is a novel pathway and module enrichment analysis algorithm designed specifically for untargeted metabolomics. It uses metabolomic features as input and thereby it does not require metabolite identification a-priori. The detailed methods can be found in Li et al. (2013). Briefly, since the computational prediction of metabolites from spectral peaks often results in multiple possibilities, a “null” distribution can be estimated by how these predicted metabolites from a metabolomics experiment map to all known metabolite reactions. The enrichment pattern of metabolites associated with the outcomes is then compared to the null distribution. False annotations are minimized, since the biological meaning in the data drives enrichment of metabolite subsets. Thus, mummichog can predict significant pathways and network modules directly from untargeted metabolomics data. The enriched pathways inferred by the algorithm have been previously validated to reflect real biological activity in numerous previous studies. Features previously selected by PLS-DA VIP > = 2 were included in the pathway enrichment analysis. Pathways were considered as being statistically significantly enriched if gamma-adjusted *p*-values were smaller than 0.05 (Uppal et al., 2015). We only reported pathways with a size of at least 3 metabolites per pathway detected. We performed a sensitivity analysis among children born at term only (gestational age > = 37 weeks) to determine whether there were similar perturbated pathways present among term birth neonates.

## Results

The demographic characteristics of the 883 subjects included in this study are shown in Table 1. Compared to non-smoking mothers, smokers tended to be more often White non-Hispanic (48.4 vs. 30.8%), aged 25–34 years when the index child was born (56.9 vs. 49.7%), foreign born (62.7 vs. 52.1%), and high school graduates (61.3 vs. 49.0%). Children whose mothers were smokers were less likely to be firstborn (35.9 vs. 42.3%) and born after the year 2000 (15.7% vs. 44.9%), while they were more likely to be male (56.9 vs. 47.4%) and preterm births (11.9 vs. 9.5%).

**Table 1:** Demographic characteristics of the study population

	Non-smoker (*N* = 730)	Smoker (*N* = 153)
	*N* = 883	
*Maternal race/ethnicity*		
White non-Hispanics	225 (30.8%)	74 (48.4%)
Hispanic of any race	370 (50.7%)	49 (32.0%)
Other/not specified	135 (18.5%)	30 (19.6%)
*Birth Year*		
1983–1990	121 (16.6%)	63 (41.2%)
1991–2000	281 (38.5%)	66 (43.1%)
2001–2011	328 (44.9%)	24 (15.7%)
*Sex*		
Male	346 (47.4%)	87 (56.9%)
Female	384 (52.6%)	66 (43.1%)
*Maternal age*		
< 20	81 (11.1%)	18 (11.8%)
20–24	181 (24.8%)	38 (24.8%)
25–29	193 (26.4%)	44 (28.8%)
30–34	170 (23.3%)	43 (28.1%)
35 +	105 (14.4%)	10 (6.5%)
*Census-based neighborhood SES index*		
1 (Low)	163 (22.3%)	30 (19.6%)
2	208 (28.5%)	40 (26.1%)
3	145 (19.9%)	47 (30.7%)
4	117 (16.0%)	18 (11.8%)
5 (High)	97 (13.3%)	18 (11.8%)
*Parity*		
0	309 (42.3%)	55 (35.9%)
1	226 (31.0%)	38 (24.8%)
2 +	194 (26.6%)	60 (39.2%)
Missing	1	0
*Preterm birth*		
Preterm	67 (9.5%)	17 (11.9%)
Term	638 (90.5%)	126 (88.1%)
Missing	25	10
*Maternal education*		
Less than high school	82 (12.5%)	10 (9.0%)
High school graduate	321 (49.0%)	68 (61.3%)
Some college, college graduate or more	252 (38.5%)	33 (29.7%)
Missing	75	42
*Foreign-born*		
Yes	380 (52.1%)	96 (62.7%)
Missing	0	1

**Fig. 1:**
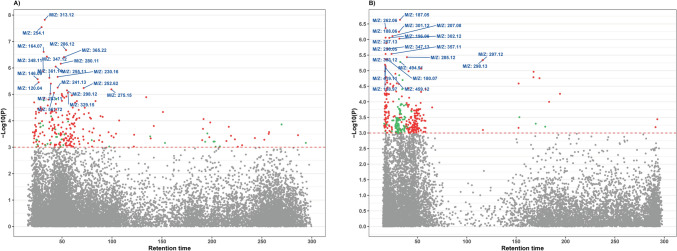
Identification of metabolic features associated with maternal smoking exposure among studied infants. **A** Type 2 Manhattan plot for features in the HILIC column (positive ion mode), − log10(*p*-value) vs retention time. Red dots represent features that were positively associated with maternal smoking exposure and green dots represent features that were negatively associated with maternal smoking exposure. *P*-values were derived from the logistic regression. Mass-to-charge ratio (*m/z*) of the important features (top 20) were labeled; **B** Type 2 Manhattan plot for features in the C18 column (negative ion mode), − log10(*p*-value) vs retention time. HILIC, Hydrophilic Interaction Liquid Chromatography; *m/z*, mass-to-charge ratio; C18, C18 Hydrophobic Reversed-Phase Chromatography

**Fig. 2:**
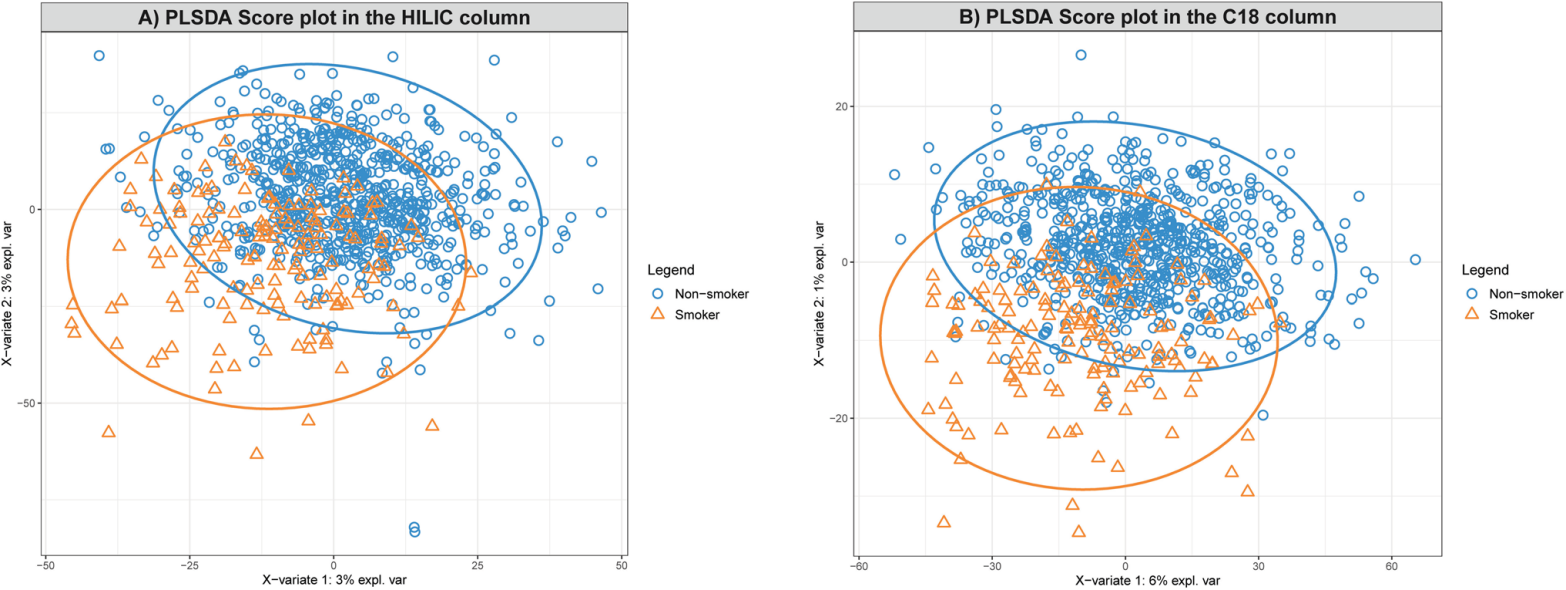
PLS-DA score plots illustrating the separation of metabolic profiles by maternal smoking status. **A** Score plot for the HILIC column in positive ion mode. **B** Score plot for the C18 column in negative ion mode. Each point represents an individual newborn’s metabolic profile, with blue circles indicating non-smokers and orange triangles indicating smokers. The ellipses represent the 95% confidence regions for each group, showing the separation of metabolic profiles between smokers and non-smokers. X-variate 1 and X-variate 2 denote the top two principal latent variables, with the percentage of explained variance displayed along the axes. HILIC, Hydrophilic Interaction Liquid Chromatography; C18, C18 Hydrophobic Reversed-Phase Chromatography

In total, we detected 26,183 features (15,562 in HILIC column and 10,621 in C18 column) with missing values in less than 30% of the samples. After discriminant analysis, we observed 520 HILIC features and 483 C18 features as being statistically significantly associated with maternal smoking where their PLS-DA VIP score > = 2. Figure 1 shows metabolic features significantly associated with maternal smoking exposure in each column (logistic regression − log10 (*p*-value) > 3; *p* < 0.001), with the top 20 features labeled by mass-to-charge ratio (*m/z*). These findings highlight distinct metabolic alterations linked to maternal smoking exposure in newborns. Score plots (Fig. 2) show how the PLS-DA analysis effectively differentiates features by smoking status.

Among the discriminative features, we confirmed the identity of 17 metabolites using authentic standards with identification confidence level 1 (Schymanski et al., 2014) (Table 2). The pathway enrichment analysis selected 10 pathways that were associated with maternal smoking exposure (Table 3). Sensitivity analysis in which we restricted to children born at term corroborated the identification of vitamin A (retinol) and androgen and estrogen biosynthesis pathways and identified other amino acid-related pathways (see Supplemental Table 2). We listed the annotated metabolites within each enriched pathway in the Supplemental Table 1.

**Table 2:** Confirmed chemical identity of metabolic features associated with maternal smoking status among studied infants

m/z	RT (s)	Adduct Form	Metabolite	Coefficient^a^	FDR^a^	VIP	Mode
90.055	52.4	M+H	Alanine	2.90E-01	2.93E-03	2.23	HILIC
102.055	66.2	M+H	1-Aminocyclopropane-1-carboxylate	3.41E-01	1.22E-03	2.11	HILIC
116.0344	78.1	M+H	Maleamate	3.25E-01	1.33E-03	2.15	HILIC
132.0766	53.7	M+H	Creatine	2.51E-01	1.03E-02	2.01	HILIC
146.0924	25.7	M+H	Guanidinobutanote	5.40E-01	8.05E-05	2.85	HILIC
146.1176	36.4	M+H	Acetylcholine	4.75E-01	1.27E-04	2.73	HILIC
180.0653	44.2	M+H	Hippurate	3.17E-01	1.45E-03	2.20	HILIC
209.0922	51	M+H	Kynurenine	3.89E-01	2.11E-04	2.86	HILIC
241.0308	183.1	M+H	Cystine	-3.36E-01	8.27E-04	2.34	HILIC
269.2263	22.4	M+H	Vitamin A (Retinol)	3.73E-01	9.35E-04	2.29	HILIC
129.0187	22.6	M−H	Itaconate	4.29E-01	1.37E-04	2.33	C18
180.0667	19.6	M−H	L-Tyrosine	4.22E-01	1.37E-04	2.18	C18
188.0566	17.8	M−H	N-Acetyl-Glutamic Acid	5.32E-01	2.93E-05	2.60	C18
195.0512	18.8	M−H	Gluconic Acid	3.46E-01	2.82E-04	2.72	C18
309.2803	291.2	M−H	FA 20:1 (Gondoic acid)	3.26E-01	3.76E-04	2.72	C18
311.296	291.3	M−H	Arachidic Acid	2.47E-01	1.14E-03	2.63	C18
588.076	17.2	M−H	Adenosine-5’-Diphosphoglucose	4.02E-01	2.06E-04	2.20	C18

Chemical identification was conducted by matching peaks by accurate mass and retention time to authentic reference standards in an in-house library run under identical conditions using tandem mass spectrometry. These metabolites are confirmed with identification confidence level 1 (Schymanski et al., 2014)

^a^Coefficients and adjusted *p*-values (FDR) were derived from logistic regression, adjusted for potential confounders. Positive coefficients mean the metabolite is higher among smokers; negative coefficients mean the metabolite is lower among smokers.”

HILIC, Hydrophilic Interaction Liquid Chromatography; C18, C18 Hydrophobic Reversed-Phase Chromatography; *m/z*, mass-to-charge ratio; RT, retention time; FDR, false-discovery rate; VIP, Variable Importance in Projection

**Table 3:** Enriched metabolomic pathways associated with maternal smoking status among all infants (*N* = 883)

Pathway	Overlap size	Pathway size	Gamma-adjusted *p*-value^a^	Mode
Vitamin A (retinol) metabolism	4	24	0.00992	HILIC
Tryptophan metabolism	7	73	0.02596	HILIC
Arachidonic acid metabolism	4	33	0.03218	HILIC
C21-steroid hormone biosynthesis and metabolism	5	49	0.03823	HILIC
N-Glycan biosynthesis	3	23	0.04579	HILIC
Vitamin A (retinol) metabolism	3	22	0.01622	C18
Androgen and estrogen biosynthesis and metabolism	4	40	0.0184	C18

^a^The gamma-adjusted *p*-value was calculated by resampling from the reference list. The *p*-values from resampling are modeled as a Gamma distribution, and the EASE scores (Hosack et al., 2003) of real data are converted to adjusted *p*-values on the CDF (cumulative distribution function) of the Gamma model. The EASE score is a variant of Fisher exact test after removing one hit in the pathway analysis. Since this is a resampling and permutation-based approach, the *p*-value calculation is not making any assumptions about the pathways being independent or not correlated with each other

## Discussion

Our study is one of the first to examine metabolic alterations in newborns due to maternal perinatal smoking using a large population-based sample of California children born over almost three decades ago. High-resolution metabolomic profiling methods allowed us to identify newborn blood metabolome alterations due to tobacco smoke exposure and our results suggest the involvement of vitamin A (retinol), tryptophan, and arachidonic acid metabolism pathways, as well as androgen and estrogen biosynthesis and metabolism. Some of these pathways such as the kynurenine pathway and tryptophan metabolism have previously been associated with an increased risk of chronic disease like lung cancer and neurological diseases.

We identified higher levels of vitamin A (retinol) in infants of smoking mothers and the vitamin A (retinol) metabolism pathways were enriched in both HILIC and C18datasets. Retinoids, which include retinol, retinoic acid, and retinyl ester, regulate biologic processes and play a role in the prevention and treatment of several chronic diseases, including cancer (Mernitz & Wang, 2006). Retinol and retinyl esters are the most abundant forms of retinoids present in the body and originate either directly from diet or are produced in the body through enzymatic cleavage of specific carotenoids (Mernitz & Wang, 2006; O’Byrne & Blaner, 2013). Lower levels of retinol were associated with declined pulmonary function (Schünemann et al., 2001). Cigarette smoking exposure has been associated with the reduction of retinoic acid and may be responsible for an increased lung cancer risk in rats (Xue et al., 2015). In addition, retinoic acid is crucial for regulating immune system homeostasis and for inflammatory responses (Erkelens & Mebius, 2017). We observed an alteration in vitamin A metabolism in newborn blood as a metabolic consequence of maternal tobacco smoking, suggesting a possible upregulation to counteract inflammatory actions.

Consistent with the existing literature, our results indicate an association between maternal smoking and elevated kynurenine in newborn blood as well as with tryptophan metabolism. Tryptophan is an essential amino acid used to build proteins and is a biosynthetic precursor for many neurologically active compounds. The kynurenine pathway plays a vital role in tryptophan metabolism and accounts for 95% of all tryptophan metabolism (Davis & Liu, 2015; Zhu et al., 2021). Activation of the tryptophan metabolism via the kynurenine pathway prevents hyperinflammation and has immunosuppressive effects. It also regulates energy homeostasis, brain function and helps regulate the mothers’ immune system during pregnancy (Broekhuizen et al., 2021; Sorgdrager et al., 2019a). Tryptophan/kynurenine metabolism has been linked with several diseases related to smoking. For example, a nested case–control study of 5364 smoking-matched case–control pairs found that those measuring in the highest quintile of kynurenine were at a 20–30% higher risk of developing lung cancer with the strongest associations seen for current smokers, lesser among former smokers, and none among never smokers (Huang et al., 2020). Another study identified tryptophan metabolism to be associated with both cotinine level and adverse birth outcomes such as shorter gestations (Tan et al., 2022). The kynurenine pathway has also been implicated in the pathophysiology of a range of neurodegenerative diseases including multiple sclerosis, Amyotrophic Lateral Sclerosis, Huntington’s and Parkinson’s disease, and Alzheimer’s disease (Lovelace et al., 2017). Low serum tryptophan can also contribute to immunodeficiency (Schröcksnadel et al., 2006). On the other hand, study results have been inconsistent with some finding a decreased level of kynurenine in smokers relative to non-smokers (Naz et al., 2019). Inconsistent findings may be due to the nature of the disease investigated (Naz et al., 2019; Sorgdrager et al., 2019b) or lifestyle factors and medications that are related to the disorder under investigation.

We identified several other tobacco smoking-related pathways and metabolites that were previously mentioned in the literature in connection with smoking. Arachidonic acid metabolites and enzyme transcripts involving both the lipoxygenase and cyclooxygenase pathways were reported to have different concentrations in smokers with asthma compared with never smokers, in multiple types of samples including urine, sputum, and blood (Thomson et al., 2014). Alanine aminotransferase is widely used as a clinical biomarker of hepatic health as it is involved in the transamination of alanine and presents in substantially higher concentrations in the liver than other organs (Gwaltney-Brant, 2016). Smoking has been shown to be associated with elevated alanine aminotransferase levels among anti-hepatitis C virus antibody-seropositive individuals (Wang et al., 2002). Hippurate has also been shown to be increased with exposure to byproducts of cigarette smoke, such as phenolic compounds and toluene, in a study examining cigarette smoking associated vaginal tract metabolomic profile (Nelson et al., 2018). Itaconate was reported to be significantly increased with cigarette smoking and has been implicated as an immune-response regulator in macrophages in a multi-omics study (Titz et al., 2020).

Our sensitivity analysis that excluded preterm births infants found pathways previously identified in a study that investigated maternal light active smoking and secondhand smoke exposures in amniotic fluid and maternal serum (Fischer et al., 2017). These pathways affected by maternal smoking exposure include vitamin A (retinol), lysine, tyrosine, porphyrin, and urea cycle/amino group metabolism. Smoking is a known risk factor for preterm birth (Carter et al., 2019) and the metabolic perturbations observed in the whole population but not in term birth may involve pathways related to preterm delivery. Out of the seven enriched pathways we identified as being different in smokers in analyses that included preterm births, three pathways (tryptophan metabolism, arachidonic acid metabolism, and steroid hormone biosynthesis and metabolism) were previously reported as pathways predictive of preterm birth (Carter et al., 2019).

Previous studies have investigated tobacco metabolism and related pathways in adults, including pregnant women, and children, but not newborn infants. Our study affirms that several pathways previously identified in adults and children are also perturbed in newborn infants whose mothers smoked in pregnancy. For example, the vitamin A (retinol) metabolism pathway, which plays a role in the development of the immune system, was reported to be altered by tobacco smoke exposure based on both second-trimester amniotic fluid and corresponding maternal serum analyses (Fischer et al., 2017). Similar to our findings, previous studies also identified kynurenine, a part of tryptophan metabolism, as smoking-related metabolites in adult smokers’ serum and children’s urine samples after secondhand smoke exposure (Xu et al., 2013; Zhu et al., 2021). Furthermore, five metabolic pathways (aspartate and asparagine metabolism and pyrimidine urea cycle/amino group metabolism, arginine and proline metabolism, and xenobiotics metabolism) reported as being associated with tobacco smoke exposure in both second-trimester amniotic fluid and in adult serum of military personnel (Fischer et al., 2017; Jones et al., 2016), were not seen to be enriched in our study. The differences in findings across studies may be due to sample type and age, as well as differences in other demographic cofactors that influence metabolism and the kinetics of nicotine metabolism (Hukkanen et al., 2005). It has been suggested that neonates have a much slower nicotine metabolism such that nicotine has a 3 to 4 times longer half-life in newborns compared with adults (Hukkanen et al., 2005). Cotinine, on the other hand, is reported to have a similar half-life in neonates, older children, and adults of about 16.3 h (95% CI 12.4 to 23.9) in blood and 22.8 h (95% CI 19.5 to 25.8) in urine (Dempsey et al., 2000; Hukkanen et al., 2005). Despite having slightly longer half-life, this biomarker derived measure from neonatal blood spots is nevertheless imperfect and may underestimate maternal smoking due to a number of reasons such as women not initiating breastfeeding (He et al., 2023a). The metabolic alterations we identified in this study in newborn blood may only reflect those metabolic pathways most strongly associated with chronic pre-and perinatal tobacco smoke exposure.

We acknowledge several limitations of this study. First, we were only able to control for potential confounders that are reported on birth certificates and with relatively low missingness. For example, we decided not to adjust for maternal and paternal education and BMI due to the large missingness of these variables. Second, due to the nature of untargeted metabolomics, the lack of breastfeeding information, and the impact of the timing of heel prick blood spot collection, we cannot obtain the absolute concentration of cotinine as a quantifiable measure of current smoking status. Instead, we utilized cotinine and hydroxycotinine and selected a plausible cutoff according to reported maternal smoking in California during the years of the study. Thus, we might have misclassified exposure status, however, this would have been non-differential for exposure and the metabolomic profile. Our classification of smokers favored high specificity over sensitivity, therefore we may have called some smokers non-smokers. We would not have been able to identify tobacco metabolites in all exposed infants if mothers did not smoke within 20 h of sample collection, and in particular among neonates of mothers who never initiated breastfeeding. Nevertheless, we were able to identify a number of metabolites and pathways that were strongly associated with tobacco smoke and have been reported previously in relation to smoking. Another limitation is the limited ability to annotate and confirm metabolites using the untargeted platform. We were able to improve the coverage range and accuracy of the annotation by adopting pathway analysis. While we used a database of confirmed standards to identify some metabolites, compounds not present in this database were annotated computationally. Although there may still be incorrect matches, our conservative annotation strategy is expected to minimize impact on interpretation, and concurrence with prior research in adults and children supports our findings.

In conclusion, we identified metabolites and pathway perturbations that have previously been associated with cigarette smoking, inflammatory responses, and diseases of the central nervous system and the lung in neonatal blood. Our results provided a global view of the newborn infant’s metabolism in response to maternal smoking in the perinatal and early postnatal period. Our study suggests that maternal smoking during pregnancy has an impact on the child’s metabolism during development and implicates pathways related to diseases previously associated with smoking such as lung disease and brain development.

## Supplementary Information

Below is the link to the electronic supplementary material.

Supplementary file1 (XLSX 39 KB)

## Data Availability

Data availability is under the regulation of the California Committee for the Protection of Human Subjects.
